# MCT2 overexpression promotes recovery of cognitive function by increasing mitochondrial biogenesis in a rat model of stroke

**DOI:** 10.1080/19768354.2021.1915379

**Published:** 2021-04-23

**Authors:** Xiaorong Yu, Rui Zhang, Cunsheng Wei, Yuanyuan Gao, Yanhua Yu, Lin Wang, Junying Jiang, Xuemei Zhang, Junrong Li, Xuemei Chen

**Affiliations:** a Department of Neurology, The Affiliated Jiangning Hospital with Nanjing Medical University, Nanjing, People’s Republic of China; b Department of General Practice, The Affiliated Jiangning Hospital with Nanjing Medical University, Nanjing, People’s Republic of China

**Keywords:** Stroke, MCT2, mitochondrial biogenesis, cognitive impairment

## Abstract

Monocarboxylate transporter 2 (MCT2) is the predominant monocarboxylate transporter expressed by neurons. MCT2 plays an important role in brain energy metabolism. Stroke survivors are at high risk of cognitive impairment. We reported previously that stroke-induced cognitive impairment was related to impaired energy metabolism. In the present study, we report that cognitive function was impaired after stroke in rats. We found that MCT2 expression, but not that of MCT1 or MCT4, was markedly decreased in the rat hippocampus at 7 and 28 days after transient middle cerebral artery occlusion (tMCAO). Moreover, MCT2 overexpression promoted recovery of cognitive function after stroke. The molecular mechanism underlying these effects may be related to an increase in adenosine monophosphate-activated protein kinase-mediated mitochondrial biogenesis induced by overexpression of MCT2. Our findings suggest that MCT2 activation ameliorates cognitive impairment after stroke.

## Introduction

Stroke is a leading cause of mortality and long-term disability worldwide and is associated with significant treatment and post-stroke care costs (Adoukonou et al. 2020). Stroke patients often have cognitive deficits due to damage of the hippocampal and cortical regions of the brain (Delattre et al. 2018). Although understanding of the molecular basis and mechanisms underlying the cerebral ischemic cascade have improved, therapeutic strategies for the treatment of post-stroke cognitive deficits remain limited. Thus, effective treatments for cognitive decline after stroke are urgently needed.

Stroke survivors are at high risk of cognitive impairment within 3 months of the stroke (Nakling et al. 2017). The risk factors for cognitive impairment include age, smoking, hyperlipidemia, hypertension, and diabetes mellitus (Kim et al. 2017; Koyanagi et al. 2018; Meguro and Dodge 2019; Wong et al. 2019; Xiu et al. 2019); however, the precise mechanism underlying post-stroke cognitive impairment remains unclear. Vascular cognitive impairment and Alzheimer’s disease (AD) are the main causes of post-stroke cognitive decline (Bordet et al. 2017). Several neurological diseases are associated with decreased energy metabolism in the brain, which further impairs cognitive function (Di Domenico et al. 2017; Jha and Morrison 2018). Previous studies have shown that cognitive impairment caused by AD may be related to mitochondrial damage, and that mitochondrial quality control is essential for maintaining cellular energy metabolism (Abolhassani et al. 2017).

Lactate and pyruvate are the major tricarboxylic acid cycle substrates (Hui et al. 2017). Monocarboxylate transporters (MCTs) facilitate lactate efflux in glycolytically active cells to maintain monocarboxylate homeostasis and intracellular pH (Zhang et al. 2020). MCT2, a major monocarboxylic acid transporter, is involved in the transport and metabolism of lactate (Lund et al. 2018). MCT2 plays an important role in cellular energy metabolism (Harun-Or-Rashid et al. 2020). Recent studies have shown that neurodegenerative diseases, such as Parkinson’s disease (PD) and AD, are related to impaired energy metabolism (Kori et al. 2016; Agnihotri and Aruoma 2020). A previous study found that MCT2 expression was reduced in several neurological diseases (Bosshart et al. 2020; Felmlee et al. 2020); however, it remains unclear whether MCT2 activation can prevent or treat stroke. We assessed changes in the expression of MCT2 in a rat model of stroke to determine whether it is possible to improve stroke-induced cognitive impairment. We further investigated the effect of molecular mechanisms on cognitive function by altering the expression of MCT2.

## Materials and methods

### Animals

Male Sprague–Dawley (SD) rats (200–240 g; 6 weeks of age) were obtained from Charles River Laboratory Animal Facility (Beijing, China). All animals were housed in a temperature- and humidity-controlled environment under a 12-h light/dark cycle with standard mouse chow and water available *ad libitum*. All animal procedures were approved by the Animal Research Ethics Committee of Nanjing Medical University (Nanjing, China) and the Laboratory Animal Management Committee of Jiangsu Province (Table 1.

**Table 1.** The primers used for real-time quantitative PCR.

Gene		5′-3′
MCT1	Forward	GACGGCAGAAGTACAAACGG
	Reverse	CAACCAGCAGATGCGACAC
MCT2	Forward	AGCACGGAGTGACCCAAAC
	Reverse	TGTACGTGGCTACATGGACCT
MCT4	Forward	AGGTGTGGTGCCAGATCTTC
	Reverse	GGACTCCTATGTGGGTGACG
PGC-1α	Forward	TATGGAGTGACATAGAGTGTGCT
	Reverse	CCACTTCAATCCACCCAGAAAG
TFAM	Forward	TGATTGGCACCGATCCTCG
	Reverse	CCACAGCGTCATATCATCCAG

### Animal surgery

Transient middle cerebral artery occlusion (tMCAO) was performed in SD rats as reported previously (Sun et al. 2008). Rats were anaesthetized with isoflurane. A midline neck incision was made to expose the right common carotid artery. A 3-0 polylysine-coated monofilament nylon suture was inserted into the external carotid artery and advanced into the internal carotid artery to obstruct blood flow into the middle cerebral artery (Sun et al. 2014). The filament was withdrawn after 2 h to restore blood flow. Rat body temperatures were maintained at 37.0 ± 0.5°C with warming pads and Doppler flowmetry (Moor Instruments, Essex, UK) was used to monitor cerebral blood flow during the surgical procedure.

Adeno-associated virus (AAV)-MCT2-enhanced green fluorescent protein (EGFP) (1.5 × 10^12^ vg/mL) and AAV2/9-GFP (2.1 × 10^12^ vg/mL) were purchased from Hanbio Biotechnology (Shanghai, China). AAV-MCT2-EGFP (5 µL) was injected into the right cerebral ventricle using a 10-µL Hamilton syringe 7 days after tMCAO. The injection was controlled at a rate of 0.5 µL/min, and the syringe remained in place for 5 min before being withdrawn. The stereotaxic coordinates of the right cerebral ventricle were bregma, 0.8 mm; lateral, 1.5 mm; and ventral, 4.0 mm.

### Behavioral assessment

#### Corner test

The corner test was performed to assess sensorimotor deficits as described previously (Sun et al. 2019). The apparatus consisted of two transparent boards placed together at a 30° angle. The rat was placed between the boards facing the corner. As the rat approached the corner, both sides of the vibrissae were stimulated causing it to turn back to face the open end. Non-ischemic rats turned to the right or left randomly, whereas the tMCAO rats preferably turned toward the non-injured side. A 15-s rest period was included between each trial and turns toward the non-impaired side were recorded.

#### Novel object recognition task

In session one, the rat was placed in a rectangular arena and exposed to two identical objects (two red cylinders) as the familiar object. In session two, which was conducted 1 h after session one, the rat was exposed to the familiar object (red cylinder) and a novel object (white rectangular box) for 5 min. The time spent exploring the familiar and novel objects was recorded to obtain the discrimination index (DI) calculated as DI = (time exploring the novel object – time exploring the familiar object)/(total amount of time exploring the novel and familiar objects) (Cole et al. 2019).

#### Morris water maze

The Morris water maze, which is widely used to assess spatial learning and memory, was performed as described previously (Shahidi et al. 2004). The Morris water maze task was performed between 22 and 28 days after tMCAO. During the visible platform tests (test days 1 and 2), a cylindrical, white platform (10-cm in diameter) was placed 1 cm above the water surface. During the hidden platform tests (test days 3–5), the platform was lowered 1–2 cm under the surface of the water. All rats were subjected to four trials per day and the swimming paths (escape latency) were recorded using a computer (AXIS-90; Neuroscience Inc., Tokyo, Japan). The average swimming time (s) and speed (m/min) to reach the visible and hidden platforms for each rat were calculated. On test day 6, the platform was removed, and the time spent in each quadrant was recorded.

### Lactate analysis

Lactate levels in the hippocampal tissue were determined using a Lactate Assay Kit (Beyotime, Shanghai, China) according to the manufacturer’s instructions. The result of the lactate assay was normalized against the protein content determined using the bicinchoninic acid method (Xu, Huang, et al. 2020).

### Adenosine triphosphate (ATP) analysis

Fresh mitochondria were isolated from hippocampal sections as previously described (Xu et al. 2020), and mitochondrial ATP content was determined using a Cell Titer-Glo® 2.0 Assay Kit (Promega, Madison, WI, USA) according to the manufacturer’s instructions.

### Immunofluorescence

At the end of the experiment, the rats were euthanized, and brain tissue was collected. The tissue was fixed overnight in fresh 4% paraformaldehyde at 4°C and dehydrated in a gradient (10, 20, and 30% mass/volume) of sucrose at 4°C and 10-µm sections were cut through the hippocampus using a Leica-1950 cryostat (Leica Instruments, Nussloch, Germany). The tissue sections were blocked with 10% normal goat serum and 3% bovine serum albumin (BSA) in phosphate-buffered saline at room temperature for 60 min and incubated with primary antibody at 4°C overnight. The primary antibody was rabbit anti-MCT2 (20355-1, Proteintech, Rosemont, IL, USA). The slides were incubated with fluorescent Alexa Fluor 488-conjugated secondary antibody (Thermo Fisher Scientific, Waltham, MA, USA) for 60 min at room temperature, and 4’,6-diamidino-2-phenylindole (DAPI, Thermo Fisher Scientific) for 5 min. The sections were viewed under a fluorescence microscope (FV1000, Olympus, Tokyo, Japan).

### Real-time polymerase chain reaction (PCR)

Real-time PCR was performed as described previously (Xu et al. 2018). Tissue samples were collected from the ipsilateral hippocampus. Total RNA was isolated from tissue using TRIzol reagent and the RNeasy Mini Kit (Vazyme Biotech, Nanjing, China). Complementary DNA (cDNA) was synthesized from 500 ng RNA using a cDNA synthesis kit (Vazyme Biotech) according to the manufacturer’s instructions. Real-time PCR was performed using the SYBR green method and gene expression was assessed using the 2^-ΔΔCT^ method (Table 1).

### Western blotting

Western blotting was performed as described previously (Xu, Zhao, et al., 2020). Tissue samples were collected from the ipsilateral hippocampus at 7 and 28 days after tMCAO and dissolved in RIPA lysis buffer (Yeasen Biotechnology Co., Ltd., Shanghai, China) containing protease and phosphatase inhibitors. Total protein (4 μg/μL, 8–10 μL/lane) was fractionated on 8% or 10% sodium dodecyl sulfate (SDS)-polyacrylamide gels and transferred to polyvinylidene fluoride membranes (Millipore, Billerica, MA, USA) by electroblotting using a miniature transfer apparatus (Bio-Rad Laboratories, Hercules, CA, USA). Next, the membranes were blocked in Tris-buffered saline containing 5% BSA for 1 h at room temperature and incubated overnight at 4°C with the following primary antibodies: rabbit anti-MCT1 (20139-1, Proteintech), rabbit anti-MCT2 (20355-1, Proteintech), and mouse anti-MCT4 (sc-376140; Santa Cruz Biotechnology, CA, USA).

### Statistical analysis

Unpaired Student’s *t*-tests were used to compare variables between groups. The remaining comparisons were assessed using a one- or two-way analysis of variance followed by Bonferroni’s multiple comparison *post-hoc* test. All statistical tests were performed using GraphPad software (ver. 8.0; GraphPad Software Inc., La Jolla, CA, USA). Data are expressed as means ± SEM. *P*-values < 0.05 were considered to indicate statistical significance.

## Results

### tMCAO-induced deficits in cognitive function in rats.

**Figure 1.**
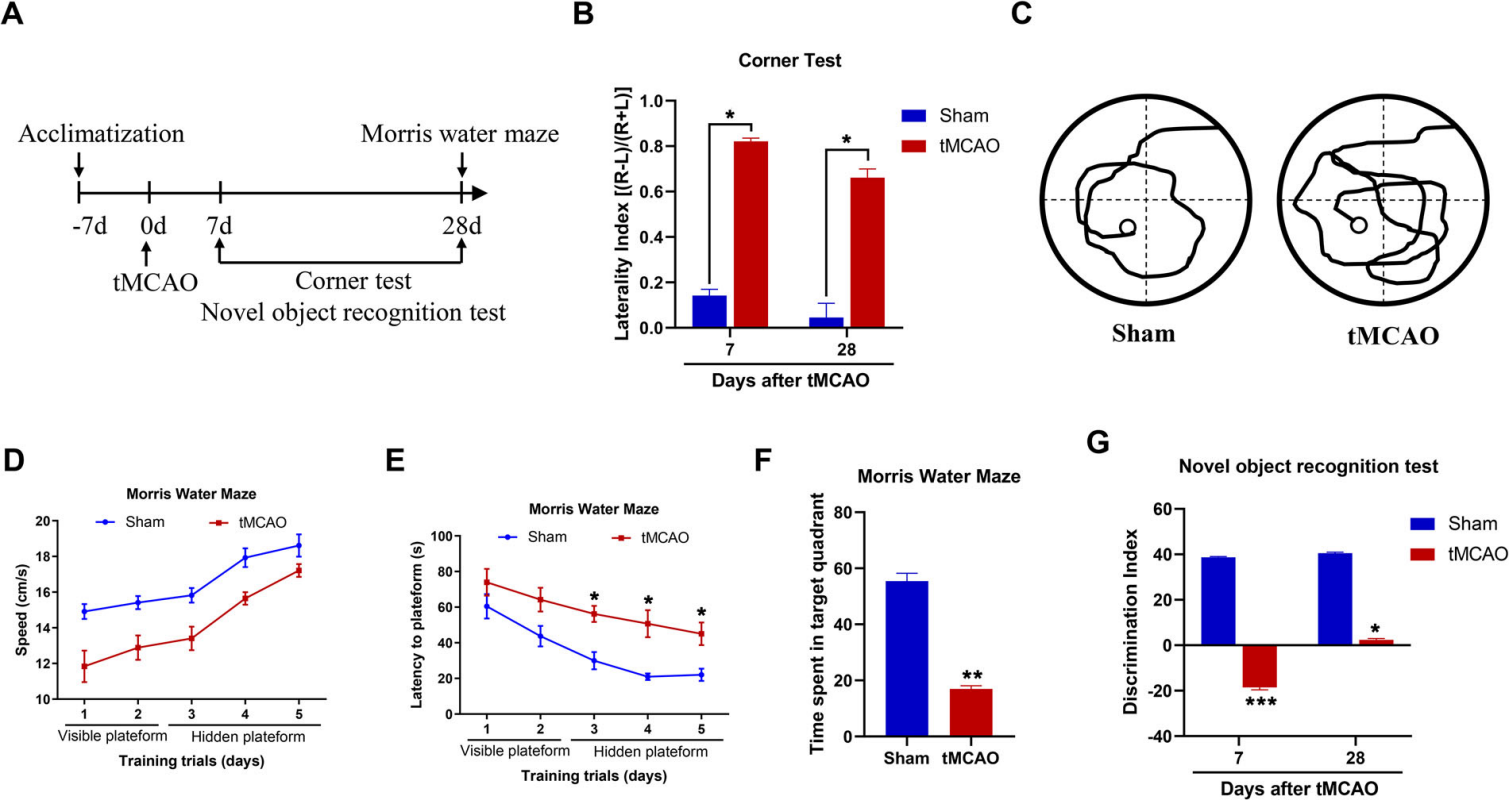
Transient middle cerebral artery occlusion (tMCAO) in rats produced deficits in cognitive function. (A) Schematic of the experimental timeline for the tMCAO surgery and behavioral tests. (B) The corner test revealed sensorimotor deficits in the tMCAO group. (C–F) The Morris water maze and (G) novel object recognition test and were used to assess cognitive function. The behavioral assessments were performed at 7 and 28 days after tMCAO. *N* = 12. All data are expressed as means ± SEM. *P < 0.05, **P < 0.01, and ***P < 0.001 vs. the sham group. A one-way ANOVA with Bonferroni’s *post-hoc* for F; two-way ANOVA with Bonferroni’s *post-hoc* for other figures.

We used the tMCAO rat model of stroke to investigate cognitive function after a stroke. The experimental protocol is shown in Figure 1A. In the corner test, the rats in the tMCAO group performed more right turns than did those in the sham group at 7 and 28 days after surgery (Figure 1B). The Morris water maze and the novel object recognition tests were used to assess cognitive function. In the Morris water maze experiment, we measured swimming speed, escape latency, and time spent in the target quadrant (Figure 1C–F). The tMCAO rats displayed slower swimming speeds and longer escape latencies, and spent less time in the target quadrant than did the sham animals. In the novel object recognition test, the rats in the tMCAO group did not discriminate between the novel and familiar objects, and their DI values were significantly lower than those of the sham group at 7 and 28 days after tMCAO (Figure 1G). These findings suggest that tMCAO produced sensorimotor, learning, and memory deficits in rats.

### MCT2 expression decreased after stroke in rats

**Figure 2.**
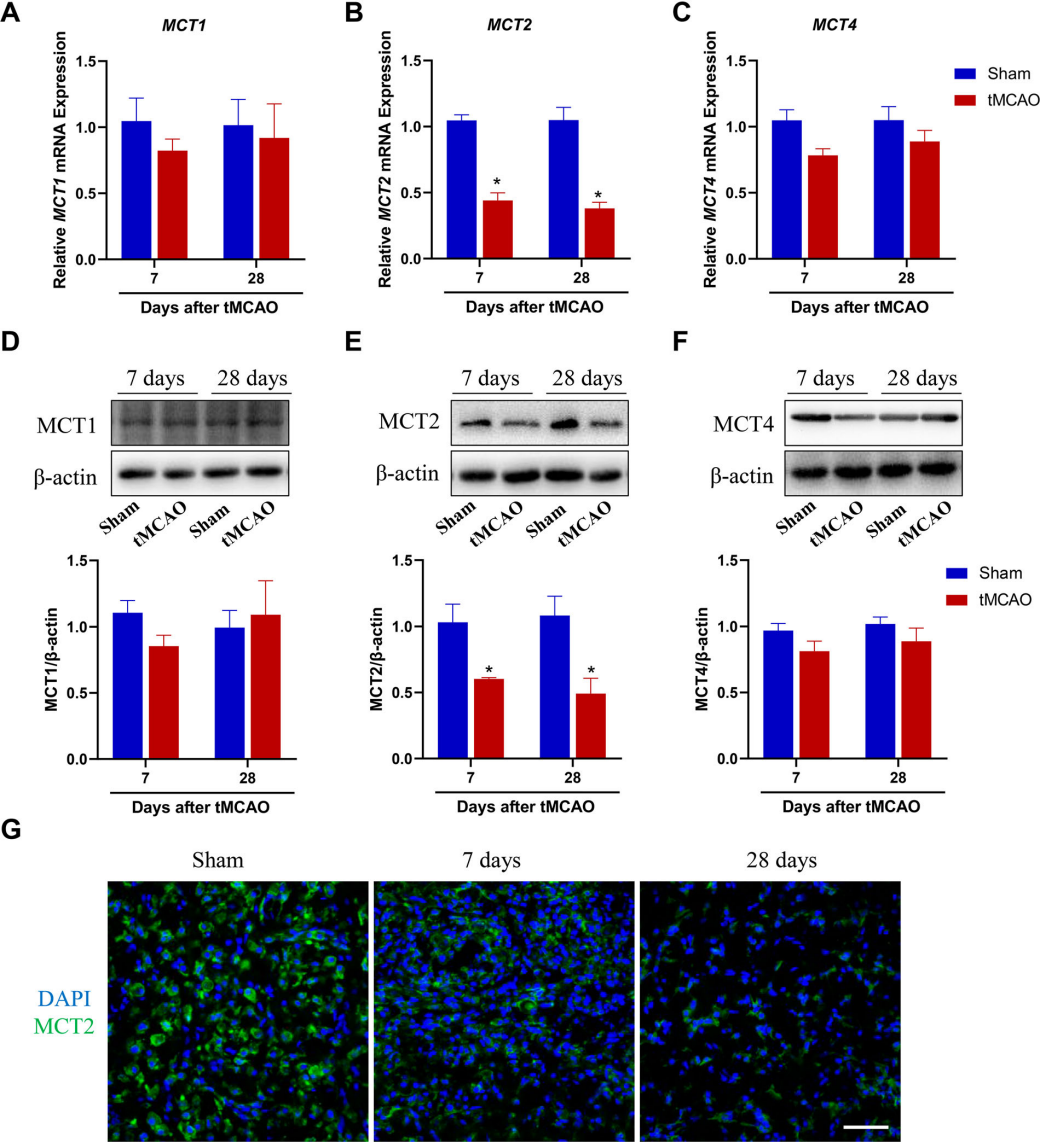
Changes in MCT2 expression after transient middle cerebral artery occlusion (tMCAO)-induced stroke in rats. (A–C) mRNA levels of MCT1, MCT2, and MCT4 detected by real-time quantitative polymerase chain reaction in the hippocampus at 7 and 28 days after tMCAO. (D–F) Western blotting of MCT1, MCT2, and MCT4 protein levels in the hippocampus at 7 and 28 days after tMCAO. (G) Representative images of tissue sections showing MCT2 (green) and 4’,6-diamidino-2-phenylindole (blue) in the ipsilateral hippocampus 28 days after ischemic stroke in rats. All data are expressed as means ± SEM. *P < 0.05 vs. the sham group. A two-way analysis of variance followed by Bonferroni’s multiple comparison *post-hoc* test.

In the absence of glucose, presynaptic terminals can metabolize lactate and lactate metabolism underlies the pathology of several diseases (Lund et al. 2018). Disruption of MCT expression has been shown to cause amnesia (Suzuki et al. 2011); however, the role of MCT in stroke remains unclear. Measurement of the mRNA levels of MCT1, MCT2, and MCT4 in the rat hippocampus after tMCAO revealed that MCT2 mRNA levels, but not those of MCT1 and MCT4, were significantly decreased at 7 and 28 days after tMCAO (Figure 2A–C). To further examine changes in MCT expression, we measured the protein levels of MCT1, MCT2, and MCT4 in the rat hippocampus 7 and 28 days after tMCAO and found that only MCT2 protein levels decreased markedly (Figure 2D–F). This finding was verified by immunofluorescence staining of MCT2 (Figure 2G). Together, these findings indicate that tMCAO decreased MCT2 expression in the hippocampus.

### MCT2 overexpression promotes the recovery of cognitive function after stroke in rats

**Figure 3.**
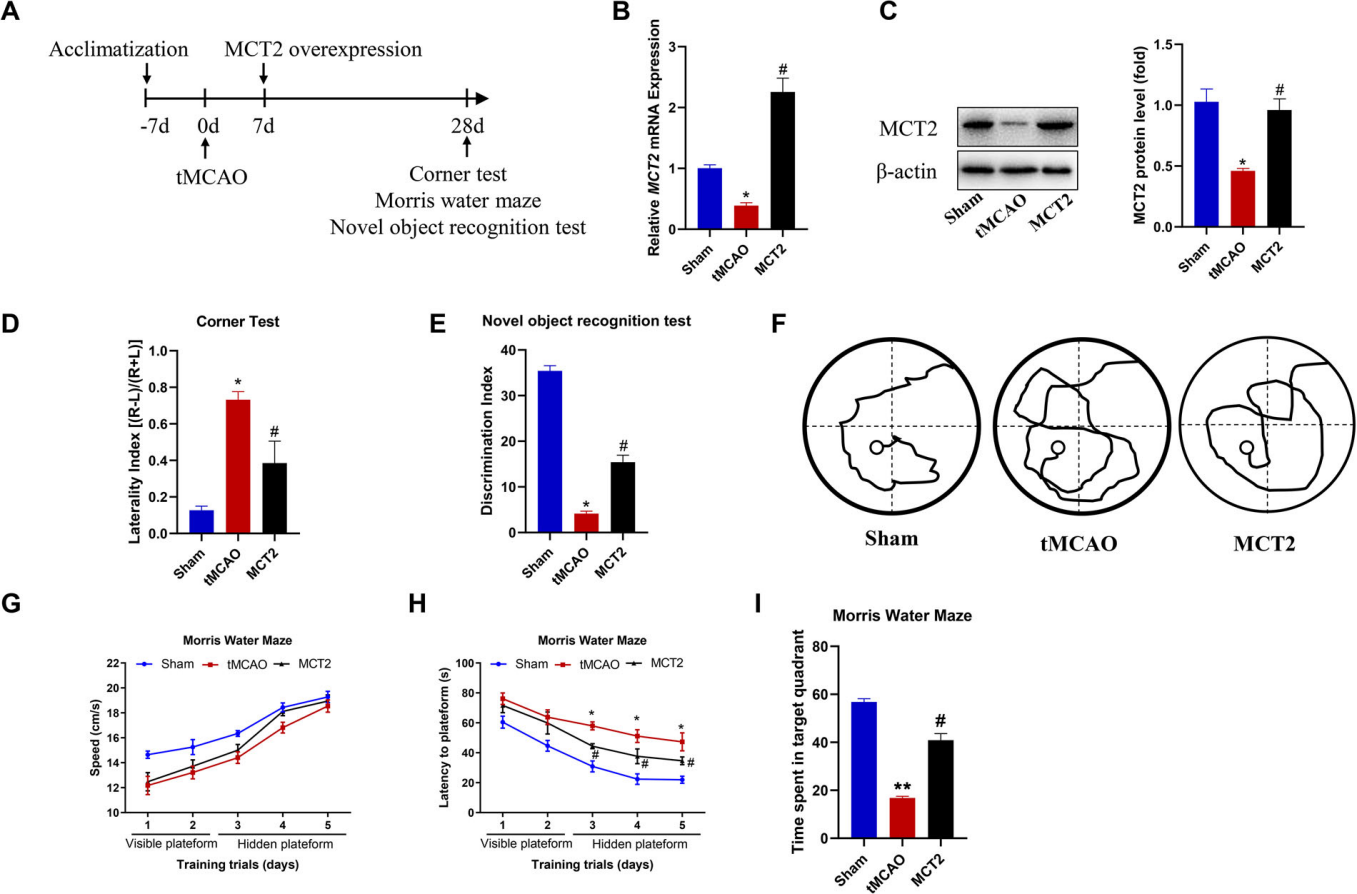
MCT2 overexpression promoted recovery of cognitive function after stroke in rats. (A) Schematic of the experimental timeline for the tMCAO surgery, induction of MCT2 overexpression, and behavioral tests. (B) MCT2 mRNA expression in the rat hippocampus. (C) MCT2 protein levels in the rat hippocampus. (D) MCT2 overexpression reduced sensorimotor deficits in the corner test. (E) The novel object recognition test and (F–I) the Morris water maze were used to assess cognitive function. The behavioral assessments were performed at 28 days after tMCAO. *N* = 12. All data are expressed as means ± SEM. #*P* < 0.05 vs. tMCAO; *P < 0.05 and **P < 0.01 vs. the sham group. A one-way ANOVA with Bonferroni’s *post-hoc* for B–E and 3I; two-way ANOVA with Bonferroni’s *post-hoc* for G and H.

While we found that MCT2 expression decreased after stroke in rats, it is not known whether MCT2 overexpression can improve cognitive function after stroke. Seven days after tMCAO, we administered AAV-2/9-MCT2 to induce, and EGFP to detect, MCT2 overexpression over a 3-week period. The experimental protocol is shown in Figure 3A. MCT2 mRNA and protein levels increased following administration of AAV-2/9-MCT2 (Figure 3B and C). In the corner test, rats in the tMCAO group performed more right turns than did those in the sham group (*P* < 0.05); this preference was reduced in rats overexpressing MCT2 (Figure 3D). In the novel object recognition test, the DI value was significantly higher in the rats overexpressing MCT2 than that in the tMCAO group (Figure 3E). In the Morris water maze test, swimming speed did not differ between the animals overexpressing MCT2 and those in the tMCAO group (Figure 3F and G). However, the escape latency time was lower (Figure 3H) and time spent in the target quadrant was greater (Figure 3I) in the MCT2 overexpression group than in the tMCAO group. These findings suggest that MCT2 overexpression improved learning and memory function after stroke.

### MCT2 overexpression increased mitochondrial biogenesis after stroke in rats

**Fig. 4:**
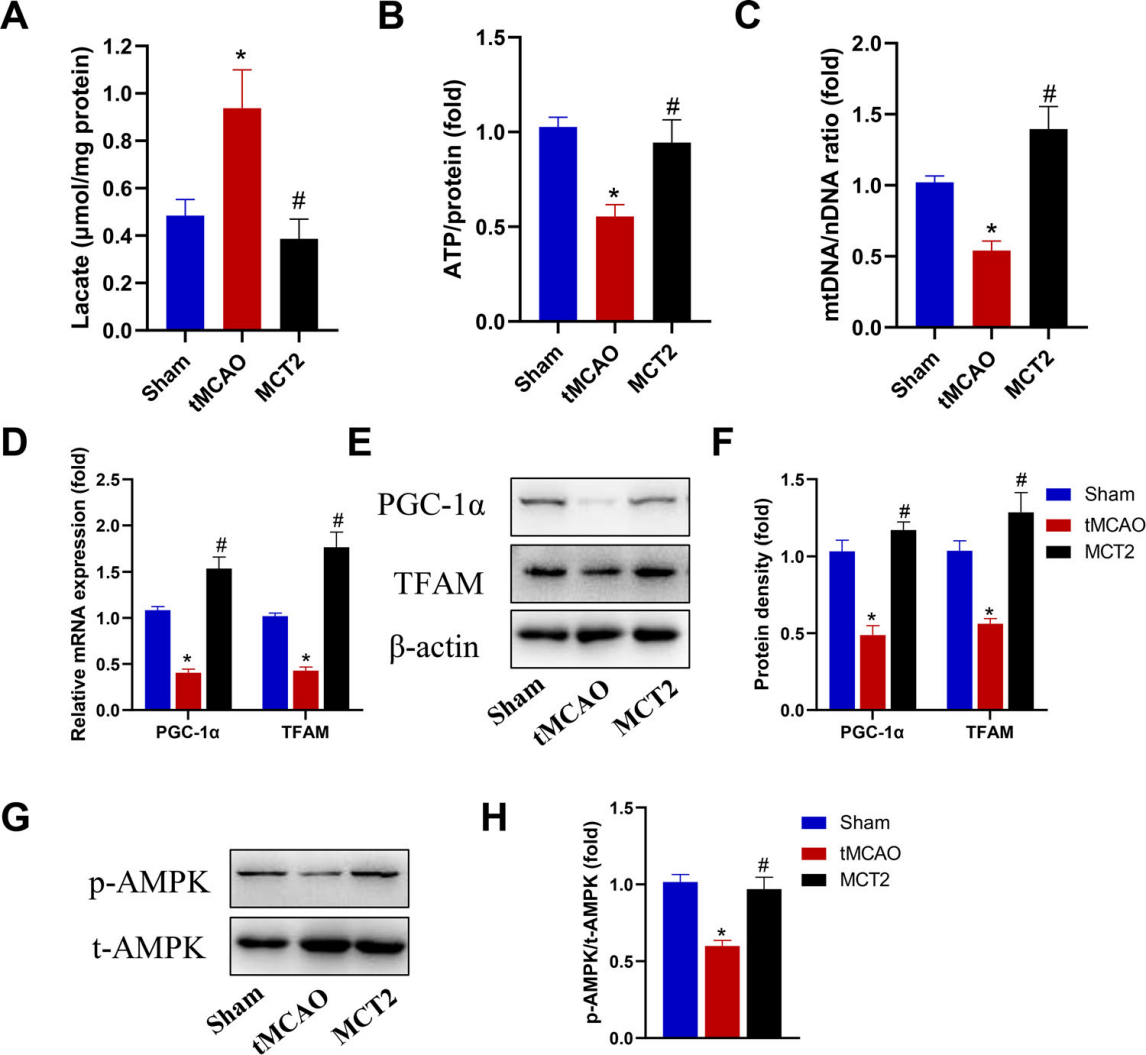
MCT2 overexpression increased mitochondrial biogenesis after stroke in rats. (A) Lactate levels, (B) ATP production, and (C) mitochondrial DNA copy number in the hippocampus after stroke in rats. (D) PGC-1α and TFAM mRNA levels were detected by real-time quantitative polymerase chain reaction. Representative images showing (E) western blotting of PGC-1α and TFAM and (F) the pooled value of each group. (G) Western blotting using anti-phospho-Thr172-adenosine monophosphate-activated protein kinase (AMPK) and anti-AMPK antibodies to determine the protein levels and (H) the ratio of phosphorylated AMPK to total AMPK (p-AMPK/t-AMPK). *N* = 12. All data are expressed as means ± SEM. #*P* < 0.05 vs. tMCAO; *P < 0.05 vs. the sham group. A one-way analysis of variance followed by Bonferroni’s multiple comparison *post-hoc* test.

We further investigated the mechanisms underlying the effect of MCT2 overexpression on cognitive recovery. Because MCT2 plays a vital role in the transport and metabolism of lactate (Zhang et al. 2005), we measured lactate concentrations in the hippocampus. We found that lactate levels were higher in the tMCAO group than in the sham group (*P *< 0.05, Figure 4A), which was reversed in animals overexpressing MCT2 (*P *< 0.05). Furthermore, ATP levels were lower in the tMCAO group than in the sham animals (*P *< 0.05, Figure 4B); however, this effect was ameliorated in rats overexpressing MCT2. These findings suggest that MCT2 overexpression promotes lactate metabolism and ATP production. Mitochondrial biogenesis is associated with ATP production and lactate metabolism. The mitochondrial DNA (mtDNA) copy number was significantly reduced in the hippocampal tissue of the tMCAO rats, and MCT2 overexpression increased mtDNA levels (*P *< 0.05, Figure 4C). The expression levels of two genes associated with mitochondrial biogenesis (*PGC-1α* and *TFAM*) decreased markedly after tMCAO; however, these decreases were reversed by MCT2 overexpression (Figure 4D). Moreover, these findings were confirmed by western blotting (Figure 4E and F). Together, our findings suggest that MCT2 overexpression increased mitochondrial biogenesis after stroke in rats.

Adenosine monophosphate-activated protein kinase (AMPK) is a key energy sensor that regulates downstream signaling pathways involved in mitochondrial biogenesis, autophagy, lipid metabolism, and glucose metabolism. Given the findings of a previous study that showed lactate suppressed AMPK activation in the brain, we measured phosphorylated AMPK (p-AMPK) and total AMPK levels in hypothalamic tissue and found that MCT2 overexpression restored p-AMPK levels (Figure 4G and H). Our findings indicate that MCT2 overexpression increases mitochondrial biogenesis via activation of the AMPK signaling pathway.

## Discussion

Recent studies have shown that neurodegenerative diseases, such as PD and AD, are associated with impaired energy metabolism (Bohnen et al. 2002; Kashiwaya and Veech 2003; Morais and De Strooper 2010). Furthermore, cognitive deficits may be due, in part, to impaired energy metabolism in the brain. Our finding that MCT2 expression was reduced in the rat brain after stroke is consistent with a previous study that found MCT2 expression was decreased in patients with AD (Pang et al. 2020). In our study, reduced MCT2 expression was accompanied by an increase in brain lactate levels. We hypothesized that cognitive impairment after stroke was due, in part, to a decrease in MCT2 expression. MCT2 is essential for lactate transport and metabolism, and our findings indicate that MCT2 activation increased AMPK-mediated mitochondrial biogenesis, thus promoting recovery of cognitive function.

MCTs are transmembrane transporters for lactate. The three MCT subtypes in the brain, MCT1, MCT2, and MCT4, synergistically transport lactate between neurons and astrocytes (Kirk et al. 2000; Fishbein and Merezhinskaya 2002). In astrocytes, MCT4 initiates the transport of lactate into the extracellular space, whereas MCT2 concurrently transports lactate into neurons (Wang et al. 2011). We found that expression of MCT1 and MCT4 in the rat brain did not change after tMCAO, whereas MCT2 levels were decreased. Moreover, we found that lactate levels in the rat brain increased after tMCAO. Our findings that MCT2 activation decreased lactic acid levels and reduced cognitive impairment support a previous study showing that lactic acid levels increased in parallel with increases in cognitive impairment.

Mitochondrial dysfunction is an underlying risk factor for stroke. Mitochondria are the powerhouses of the cell and are essential for neuronal survival and improvement in neurological function after stroke (Xu et al. 2019). Neuronal death associated with energy deficiency related to mitochondrial dysfunction causes further cognitive impairment (Morais and De Strooper 2010). In the corner test, rats in the tMCAO group performed more right turns than those in the sham group as our early report (Sun et al. 2019). The time to go to the platform take longer may partly due to decreased motor function. Therefore, we conclude that tMCAO-induced deficits in cognitive function and motor function in rats. Furthermore, previous studies have shown that mitochondria may serve as a therapeutic target for neurodegenerative diseases, including PD, ischemic stroke, and AD (Blass 1999; Petrozzi et al. 2007; Swerdlow 2009; Sudakov et al. 2010; Zhu et al. 2010). We found that activation of MCT2 improved mitochondrial function and ameliorated cognitive impairment by increasing AMPK-mediated mitochondrial biogenesis. Mitochondrial quality control is essential for the prevention of cognitive impairment; thus, targeting MCT2 may be an effective approach for ameliorating cognitive decline after stroke.

In conclusion, our study provides evidence that MCT2 expression decreases after stroke and is associated with an increase in lactate levels in the brain. MCT2 inhibition may exacerbate cognitive impairment and pharmacological activation of MCT2 may promote recovery of cognitive function by increasing AMPK-mediated mitochondrial biogenesis.

## Authors’ contributions

Xiaorong Yu, Rui Zhang and Cunsheng Wei researched the data, contributed to discussions and wrote the manuscript. Yuanyuan Gao and Yanhua Yu collected and analyzed data. Lin Wang, Xuemei Zhang and Junying Jiang reviewed the manuscript. Junrong Li conceived the experiments, researched the data and edited/reviewed the manuscript. Xuemei Chen is the guarantor of this work and, had full access to all the data in the study and takes responsibility for the integrity of the data and the accuracy of the data analysis.
